# Implicit attitudes towards transgender people: reliability and validity of a single-target implicit association test

**DOI:** 10.1016/j.ijchp.2026.100695

**Published:** 2026-05-30

**Authors:** Jacques van Lankveld, Anne Daams, Mark Hommes

**Affiliations:** Open Universiteit, Heerlen, Netherlands

**Keywords:** Transgender, Attitude, Implicit, Reliability, Validity

## Abstract

Transgender and gender diverse (TGD) individuals often experience stigma due to negative prejudices from others. Whereas research previously focused on conscious attitudes through self-reporting, more recent studies also investigate automatic, unconscious reactions. This study investigated the reliability and validity of a novel Single-Target Implicit Association Test (ST-IAT) for measuring implicit attitudes towards transgender people. In total, *N* = 85 individuals from the general population (47 *cis* men, 38 *cis* women, M_age_ = 45.6 years) participated in an online study. Based on split-half reliability analysis with Spearman-Brown correction for attenuation, the instrument was found reliable, in the sense of internally consistent (α = 0.89). Supporting the expectations with regard to its construct and predictive validity, the Transgender ST-IAT scores were positively correlated with the explicit attitude towards transgender individuals (*ρ* = 0.46, *p* < .001) and with degree of contact with transgender individuals (*ρ* = 0.25, *p* = .02). It was negatively related to genderism (*ρ* = -0.41, *p* < .001). Possibilities for future research are discussed.

## Introduction

The gender identity of transgender and gender diverse (TGD) people is not or not fully aligned with the sex assigned to them at birth (Coleman et al., 2022). The quality of life of TGD individuals is associated with a wide variety of factors, including, but not limited to, the way society at large responds to them, and their relationships in their private lives, at work, and in education (Coleman et al., 2022; Maniaci et al., 2022). It is also strongly determined by the coping of TGD people with negative responses from other people (Chinazzo et al., 2025). Compared to other people, they run a higher risk of experiencing stigmatization, violence, discrimination, and exclusion (Bos et al., 2013; Burke et al., 2025; Drabish & Theeke, 2022). Stigmatization is considered a major cause of negative health outcomes in TGD populations by inducing both acute and chronic stress (White Hughto et al., 2015). Moreover, it adds to the stress related to being member of a minority group (Frost & Meyer, 2023; Hatzenbuehler & Pachankis, 2016; Meyer, 2003). Stigma also exerts an indirect negative effect on the health quality of TGD individuals as it limits their access to health services (White Hughto et al., 2015). Understanding the determinants of discriminatory and stigmatizing behavior towards TGD people is therefore of great importance for advancing their quality of life (Hatch et al., 2022; Perez-Arche & Miller, 2021). Negative attitudes toward a stigmatized minority population (Erkal et al., 2024), and lack of contact with members of these populations (Ballinger et al., 2023; Davies et al., 2011; Yamaguchi et al., 2025) have been found to increase the likelihood of discriminatory and stigmatizing behavior in general. The mitigating effect of contact on stigmatization was also observed among transgender populations (Barbir et al., 2017; Walch, Sinkkanen et al., 2012). Another determinant for stigmatizing attitudes is *genderism,* which leans on a theoretical ‘either/or’ model of gender as a binary system that is fully biologically determined, with no possibilities for intermediate positions between both extremes or for changing positions after birth (Coleman & Hong, 2008; Gülgöz et al., 2019; Wilton et al., 2019).

Investigation of negative attitudes towards TGD people depends on both the willingness of research participants to disclose their attitudes and the extent to which such attitudes are consciously accessible. Because transgender identity is a sensitive issue and an increasing focus of polarization in some societies (Carmines & Nassar, 2021), the disclosure of one’s negative attitudes towards TGS individuals depends on the inclination of the person concerned to display socially desirable responses, as well as on the extent to which one is aware of such attitudes. These sources of potential bias increase the risk that researchers will obtain a distorted picture of attitudes toward transgender people. However, it is possible to gain access to TGD-related attitudes that are inaccessible to the person themselves or less socially acceptable by measuring people's implicit attitudes towards TGD people (Wang-Jones et al., 2018).

Implicit attitudes are not readily accessible to conscious awareness and are described as automatic, unintentional attitudes over which one has less control and of which one is less aware where they come from, what their meaning is, or what role they play (Bargh, 1994; McNally, 1995). Previous research has shown that implicit and explicit attitudes can differ from each other but can also be related (Nosek, 2005, 2007). They each explain part of the variance in social behavior (Corr, 2010; Dewitte et al., 2008; Shiffrin & Schneider, 1977; Zapata-Calvente et al., 2019), especially regarding sensitive topics (Greenwald et al., 2009).

The Implicit Association Test (IAT; Greenwald et al., 2022, 1998) is a frequently used indirect procedure for measuring implicit attitudes, based on response time differences (De Houwer et al., 2009; Gawronski & Payne, 2010; Wang-Jones et al., 2017). The IAT is considered a relative measure of implicit attitudes because it evaluates the participant's relative preference for two objects that represent different, often opposing categories. In contrast, the Single-Target Implicit Association Test (ST-IAT), a more recent adaptation of the IAT, measures the participant’s evaluation of a single target object, making it less dependent on the specific choice of a contrasting category (Bluemke & Friese, 2008; Wigboldus et al., 2004). IATs and ST-IATs have been found to both constitute reliable and valid measures of implicit attitudes (Bluemke & Friese, 2008; Greenwald et al., 2009; McNulty et al., 2014; Mechler et al., 2024).

Implicit attitudes towards TGD persons have thus far been investigated in a small number of studies (Axt et al., 2021; Wang-Jones et al., 2017, 2018), all using dual-target IATs, in which “transgender” (or “transsexual”) was contrasted with another target category. Wang-Jones et al. (2017) developed two IATs, using “transsexual men” (women) versus “biological men” (women) as target category labels, and “good” versus “bad” as attribute category labels. Preferences for ‘biological (wo)men’ were particularly prevalent among study participants who were cisgender, heterosexual, and/or politically conservative, and who had not had any personal contact with transgender people. The implicit measures did not correlate with social desirability, whereas explicit attitudes of parallel constructs did. IAT scores in this study were coded such that larger positive scores indicated greater relative preference toward cisgender over transgender targets. IAT scores correlated positively (respectively, *r* = 0.29 and *r* = 0.31) with scores on a self-report questionnaire measuring genderism and transphobia. In a follow-up study from the same lab (Wang-Jones et al., 2018), using the same IATs, gay, straight, and non-monosexual (asexual, bisexual, pansexual) participants were compared. Straight participants exhibited the strongest implicit bias toward transmen and transwomen, while non-monosexual participants showed the smallest bias. Homosexual participants displayed a positive explicit bias toward transmen similar to that of non-monosexual individuals, but at the implicit level they displayed a negative bias against transmen similar to straight monosexual respondents. With regard to the attitudes toward transwomen, implicit measurement scores were consistently negative and did not differ by group. Axt et al. (2021) developed and compared different versions of an IAT for measuring implicit attitudes toward transgender people. All IAT versions used category labels of “transgender people” and “cisgender people”. More positive IAT scores indicated more positive associations with cisgender versus transgender people. They found evidence of implicit anti-transgender attitudes, which were associated with various negative beliefs and behaviors toward transgender people, such as discrimination and gender bashing. Even after explicit attitudes were taken into account, implicit attitudes predicted outcomes related significantly to - among others - contact with transgender people (respectively, *r* = −0.20 and *r* = −0.31), and gender essentialism (respectively, *r* = 0.13 and *r* = 0.28) (Axt et al., 2021). In a study among non-healthcare professionals, nurses, and non-nursing healthcare professionals, Derbyshire and Keay (2023) used pictures of cisgender and transgender celebrities, matched for gender, skin tone, and hair style, as target stimuli, and “good” versus “bad” words as attribute stimuli. Non-healthcare professionals showed a significantly lower negative implicit bias towards transgender people, compared to healthcare professionals. Compared to non-nursing professionals, nurses exhibited higher implicit negative bias. Implicit and explicit attitudes were highly correlated.

In sum, previous work on implicit attitudes exclusively used regular, dual-target IATs. The findings in earlier studies may therefore be confounded by the choice of the contrasting category with which to compare the transgender target category, respectively, transsexual women/men with biological women/men, transgender individuals with cisgender individuals, and transgender celebrities with cisgender celebrities, as argued by Axt and colleagues et al. (2021). The term “transgender” has been found to be used three times more often than the term ‘cisgender’ in US-focused articles about health, mainly to categorize a person as “non-transgender” (Esacove, 2024). Because cisgender is a less familiar term to the general public, use of this term in a standard IAT could therefore entail a greater risk of bias (Dasgupta et al., 2000; Greenwald et al., 2022), as participants would need more time to recognize these stimuli and sort them into the “cisgender” category.

The research aims of the present study were to develop and examine the reliability and (construct and predictive) validity of a novel Single-Target IAT measuring implicit attitudes towards transgender individuals. The construct validity and predictive validity of this Dutch-language Transgender ST-IAT was investigated against explicit attitudes toward transgender people, genderism, and the degree of contact with transgender people. The following hypotheses were tested: 1. The reliability of the Transgender ST-IAT is sufficient, with split-half reliability after Spearman-Brown correction for attenuation >0.80; 2a. There is a moderate (*r* > 0.30) positive correlation between implicit and explicit attitudes toward transgender individuals (construct validity); 2b There is a moderate (*r* < −0.30) negative correlation between implicit attitude and the degree of genderism (construct validity); 2c. There is a weak (*r* > 0.20) positive correlation between implicit attitude and the frequency of contact with transgender persons (predictive validity).

## Method

### Participants

Participants in this cross-sectional study were recruited from the general Dutch population, aged 18 years or older, by email, via an online recruitment platform (Enquête Experts). Subscribers to the platform were randomly selected and invited to participate without any intervention from the researchers. No exclusion criteria were used.

### Instruments

The study consisted of four parts that were all completed within a single session: a computer task (the Transgender ST-IAT), two validated questionnaires (the Attitudes Toward Transgendered Individuals Scale (ATTI; Walch, Ngamake et al., 2012); the Genderism Scale of Dierckx et al. (2014), a brief questionnaire about contact with transgender individuals (the Contact with Transgender Individuals scale of Axt et al. (2021), and questions about demographic characteristics.

*Implicit attitude towards transgender persons*. A single-target IAT (Bluemke & Friese, 2008; Wigboldus et al., 2004) to measure participants’ implicit attitude towards transgender persons was designed, adhering to the best research practices described by Greenwald et al. (2022). The ST-IAT was administered online. We did not collect information regarding the conditions under which the implicit task was administered, including the type of device used, the setting (e.g., home or another location), the time of day, as well as potential distractions or interruptions. Although these contextual variations may have introduced unwanted variability in reaction times and may have compromised the interpretation of the results, the current approach was deemed appropriate based on previous research (Houben & Wiers, 2008).

**Fig. 1:**
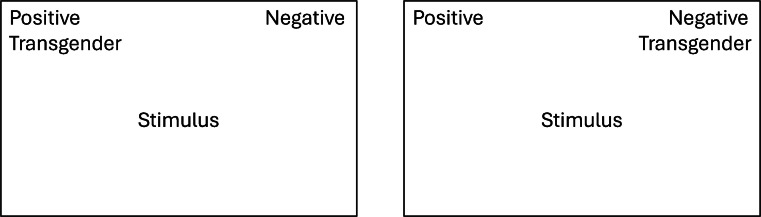
Single-target implicit association test.

In the IAT participants sorted word stimuli appearing in the center of a laptop or computer screen using two keyboard keys (a and l) on a QWERTY keyboard. The labels of the attribute categories were permanently shown in the upper-left and -right corners of the screen. The target label was shown below one of the attribute labels and switched between the left and right position in different presentation blocks, see Fig. 1. Following each response, the next stimulus was presented after a 250 ms interval. Attribute categories were ‘Positive’ (with word stimuli: smiling, happy, cheerful, kind, fun; in Dutch: lachen, blij, gelukkig, aardig, plezier) versus ‘Negative’ (with word stimuli: pain, nasty, hate, horrible, rotten; in Dutch: pijn, vies, haat, gruwelijk, verrot), and the target category was ‘Transgender persons’ (with word stimuli: trans man, trans woman, transgender person, trans, transgenderism; in Dutch: trans man, trans vrouw, transgender persoon, trans, transgenderisme). The ST-IAT was organized into five blocks: 1. Attribute practice block (20 stimuli); 2. Attribute and target practice block; 3. Test block (with target label in the same position as in block 2); 4. Attribute and target practice block (with target label in the alternate position); 5. Test block (with target label in the same position as in block 4). Practice blocks 2 and 4 each contained 20 items; test blocks 3 and 5 contained 40 items each. Blocks 2 + 3 and blocks 4 + 5 were presented to participants in randomized order to counteract possible sequence effects.

*Explicit attitude towards transgender persons.* The Attitudes Toward Transgendered Individuals Scale (ATTI; Walch, Ngamake et al., 2012) was used. The ATTI aims to measure the participant's cognitive and emotional responses to transgender individuals. This instrument was found to be internally consistent (*α* = 0.96), and evidence was shown for both convergent and divergent construct validity (Walch, Ngamake et al., 2012). The ATTI consists of 20 items, such as “It would be beneficial to society to recognize transgenderism as normal” and “Transgendered individuals should not be allowed to work with children.” Participants rated the extent to which they agreed with the statements on 5-point Likert scales (1 = strongly agree, to 5 = strongly disagree). A single-factor structure was found. The nine items that load negatively on the factor (items 1, 5, 8, 10, 12, 13, 14, 16, and 17) were reverse scored. The ATTI was translated into Dutch, back translated and checked by a native English speaker, following established procedures (Beaton et al., 2000; Tsai et al., 2018). The ATTI score was calculated by taking the average of all item scores. Higher scores indicate a more positive explicit attitude towards transgender people. The internal consistency of this scale in the present sample was excellent (Cronbach's α = 0.98). There was one missing value in the ATTI data; for this respondent, an average of the 19 other scores entered was calculated and imputed for the missing value.

*Genderism*. The Genderism scale was taken from Dierckx et al. (2014). It measures the degree of gender rigidity, whereby the participant is convinced that sex and gender are binary categories and biologically determined. Dierckx et al. compiled the scale using items from the “Trans Persons Beliefs Scale” and the “Beliefs about Gender Scale” by Tee and Hegarty (2006). The scale consists of 10 items, such as: “If you are a man or a woman, you are one for life” and “All men have a penis and all women have a vagina.” Participants rated the extent to which they agreed with the statements on 7-point Likert scales (1 = strongly agree, to 7 = strongly disagree). The internal consistency of the scale in the present sample was questionable (α = 0.62), and was lower than in the sample of Dierckx et al. (2014). After removing the question “Whether a person sees themselves as male or female is largely a matter of upbringing”, the internal consistency increased to a satisfactory level (α = 0.74). The genderism scale was calculated as the average of the remaining nine item scores. After recoding, higher scores indicate more rigid views on sex and gender, and stronger belief that these are immutable.

*Degree of contact with transgender persons*. The degree of contact with transgender persons was assessed using the scale employed by Axt et al. (2021), consisting of four questions. An example item is “Have you ever met a transgender person?” The questions could be answered with “Yes,” “No,” or “I don't know.” The scale score was calculated as a count score of the “Yes” responses. Higher scores indicate that the participant has had more contact with transgender people. Although Axt et al. (2021) reported reliability (α = 0.64) of the contact scale, it should - in our view - not be treated as a reflective measure capturing an underlying latent psychological construct, but as a formative measure, rendering examination of its internal consistence irrelevant (Ellwart & Konradt, 2011; Gruijters et al., 2021).

*Demographic data*. Finally, participants were asked to provide demographic information: age, gender assigned at birth (male/female/intersex), gender identity (male, female, transgender, non-binary).

### Ethical aspects

Ethical approval for the current study was obtained from the Research Ethics Committee of the Open Universiteit in The Netherlands. The study was checked for compliance with the following national and international regulations: Medical Research Involving Human Subjects Act, General Data Protection Regulation (GDPR), GDPR Implementation Act, Dutch Code of Conduct for Scientific Integrity, Royal Netherlands Academy of Arts and Sciences (KNAW), and Ethical Principles of Psychologists and Code of Conduct (APA).

### Procedure

Participants received a digital link to the local research platform of the Open Universiteit in The Netherlands. After reading and signing the informed consent form, they could start. Participants were randomly assigned to one of two versions in order to control for order effects within the ST-IAT. Version 1 contained the ST-IAT with block order 1-2-3-4-5, while version 2 contained the ST-IAT with block order 1-4-5-2-3. Additionally, in both versions the research components (ST-IAT and questionnaires) were presented in random order, with the demographic data and contact questions always as the last component.

### Statistical analysis

A power analysis showed that 82 participants were needed to detect an effect size of 0.30 in a two-tailed correlation analysis with a power of 80 %. The effect size estimate was based on previous studies on the associations between implicit and explicit attitudes (Axt et al., 2021; Wang-Jones et al., 2017).

For the calculation of the scores for the Transgender ST-IAT only test block data were used. An initial check was performed to identify participants who had >10 % of their responses < 300 ms, which was considered an indication of random responding. After applying this rule, the data of all participants could be retained. Response times of >10,000 ms were removed prior to calculating the average RTs. The ST-IAT index score was calculated as the difference between the average RTs of the two test blocks, divided by the pooled standard deviation of both test blocks. Higher scores indicate a more positive implicit attitude toward transgender people. Prior to analysis, the questionnaire data were screened for entry errors, missing values, and abnormal distributions and values.

Hypothesis 1 was tested using Spearman-Brown's split-half reliability analysis (cut-off value 0.80). Other hypotheses were tested using Spearman Rho correlation analyses. For all analyses, the significance level was α = 0.05. The effect sizes of the correlations were interpreted using the criteria of Schober et al. (2018).

## Results

The survey was started by 120 individuals. Only data from participants who completed all sections were included in the analysis (*n* = 85). Average age of the participants was 45.6 years (*SD* = 16.39, range = 18 to 83 years). Only cisgender women (*n* = 47, 55.3 %) and men (*n* = 38, 44.7 %) participated. Table 1 shows details of the participant’s contact with transgender people.

**Table 1:** Degree of contact with transgender persons.

	N	%
No contact with transgender persons	41	48.2
Has a transgender family member	3	3.5
Has a transgender friend	6	7.1
Has regular friendly contact with transgender people	17	20.0
Had ever (consciously) met a transgender person	42	49.4

Note: Raw N and percentages exceed, respectively, *N* = 85 and 100 %, as participants could report multiple contacts with transgender individuals.

### Reliability

The data from blocks 2 and 4 (practice blocks, each with 20 items) and the data from blocks 3 and 5 (test blocks, each with 40 items) were used to examine the internal consistency of the Transgender ST-IAT. The split-half coefficients for these blocks, as well as for the combined practice and test blocks, are shown in Table 2. The split-half reliability across all four blocks combined is 0.89, according to the Spearman-Brown approach.

**Table 2:** Spearman-Brown split-half coefficients.

	Block 2	Block 3	Block 4	Block 5	Practice blocks	Test blocks	Entire ST-IAT
Spearman-Brown coefficient	.636	.816	.723	.871	.714	.799	.888

### Validity

The assumption of normality was partially violated, with the ATTI scale and the contact scale not being normally distributed. The ATTI scale showed a left-skewed distribution, with the majority of participants reporting a positive attitude toward transgender people. The contact scale showed a right-skewed distribution, with the majority of participants knowing few transgender people or having had little contact with them. Because the assumption of a normal distribution was not met, Spearman Rho analyses for nonparametric data were performed.

The correlations between the key study variables is shown in Table 3. A moderately strong positive correlation was found between the IAT index and the ATTI scale, Spearman’s *ρ*(84) = 0.45, *p* < .001. The IAT index and the genderism scale correlated moderately negatively with each other, with Spearman's *ρ*(84) = −0.41, *p* < .001. Finally, a weak positive correlation was found between the IAT index and the contact scale, Spearman’s *ρ*(84) = 0.29, *p* = .006.

**Table 3:** Correlations of study variables.

	1	2	3
1 Implicit attitude towards transgender (ST-IAT)	-		
2 Explicit attitude towards transgender (ATTI)	.45⁎⁎	-	
3 Genderism	−0.41⁎⁎	−0.76⁎⁎	-
4 Contact with transgender persons	.29*	.11	.11

⁎ *p* < .01,.

⁎⁎ *p* < .001.

## Discussion

For the present study a novel single-target IAT was developed with the aim of measuring implicit attitudes toward transgender individuals. Its reliability, construct validity and predictive validity were tested in a small, although sufficiently large sample in terms of the required statistical power to test the study hypotheses. The sample fully consisted of cisgender women and men. The findings showed that the Transgender ST-IAT is reliable and valid. Because the Spearman-Brown coefficient of the complete ST-IAT was higher than the cutoff value (0.80), its reliability was supported. The results also supported the convergent and divergent validity of the Transgender ST-IAT, as expected based on theory and previous empirical findings. A positive correlation was found with the ATTI, suggesting that when people explicitly hold positive attitudes toward transgender individuals, they also exhibit positive implicit attitudes toward transgender individuals. In addition, a negative correlation was observed with the genderism scale, indicating that people with stronger genderist views have a more negative implicit attitude toward transgender people. The predictive validity of the Transgender ST-IAT was supported; more contact with transgender people was associated with a more positive implicit attitude towards them. These findings confirm earlier findings from research in which the contact hypothesis was tested to explain prejudice and stigmatization (Davies et al., 2011; Pettigrew, 1997).

The current findings largely corresponded with those of Axt et al. (2021), despite the fact that Axt and colleagues used different instruments to measure explicit attitude and genderism. Their study found significant correlations - conceptually in the same directions - between implicit attitude, in their case measured with a dual-target IAT, and explicit attitudes toward transgender individuals (|*r* = 0.33|), between implicit attitude and gender essentialism (|*r* = 0.13|), and the degree of contact with transgender individuals (|*r* = 0.20|). Our findings are also in line with those of Wang-Jones et al. (2017), who examined a mixed sample with regard to gender identity and sexual orientation. In their full sample, implicit and explicit attitudes towards transgender people were significantly correlated (respectively with |*r* = 0.31| for the IAT featuring transmen, and |*r* = 0.23| for the IAT featuring transwomen), conceptually in the same direction as found in the present study. In the study of Derbyshire and Keay (2023) a significant correlation was found between their IAT scores and an explicit measure of transphobic attitude. The larger effect sizes in the current study could indicate that the single-target IAT performed better as a measure of implicit transgender attitude than the dual-target IATs used for the same purpose in previous studies, but this is only speculative and awaits direct comparisons in future research.

In this study we found a moderate correlation (*ρ* = 0.45) between implicit and explicit attitudes toward transgender people. Although this correlation magnitude is somewhat higher than found on average (Hofmann et al., 2005), its meaning and implication can be interpreted in widely varying ways. One might infer from this finding that the sample under study exhibited a low degree of social desirability (Burguet & Girard, 2024) or, conversely, a high willingness to report explicit views that align with their automatic tendencies, partly because there is often some degree of awareness of one’s own implicit response tendencies (Morris & Kurdi, 2023). Alternatively, this correlation size might be taken to imply that the range restriction in the explicit measure (skewed towards positive attitudes) influences the observed relationship due to self-selection bias that is often observed in socially stigmatized domains (Burguet & Girard, 2024) and in other domains that are regarded sensitive with regard to one’s public reputation (Hofmann et al., 2005). However, it is not possible to determine the extent to which these explanations hold true based on the current data; further research is needed to test these hypotheses.

Since the study was conducted using a non-representative sample, there was an unknown degree of self-selection bias. Such bias as well as its unknown magnitude is common in research on sexuality and diversity issues (e.g., Dawson et al., 2019). In research where individuals with more favorable attitudes or greater sensitivity to the topic are more likely to participate, the findings may be biased in terms of the variability of the scores on explicit measures, such as the ATTI, which proved to be skewed towards more positive attitudes towards transgender people. This sampling bias might also decrease the instrument’s reliability (Belzak & Lockwood, 2024), the magnitude of the correlations with implicit measures (Dunlap et al., 1995) and impair the generalizability of the findings to more heterogeneous populations.

### Strengths and limitations

A strength of the current study was its use of a single-target version of the IAT. This protected the instrument from the potential consequences of the specific choice of a contrasting target category as required by the regular IAT (Bluemke & Friese, 2008; Wigboldus et al., 2004). The observed consistency of the current results with previous research using regular IATs supports the robustness of the associations between implicit transgender attitudes on the one hand, and explicit attitudes and contact on the other.

We acknowledge that this study has a number of limitations. The first concerns the entirely cisgender composition of the sample. This limits the generalizability of the findings to populations other than cisgender populations. Another limitation of this study and other studies using IAT methodology is that the implicit attitudes observed do not necessarily translate into corresponding behavior (Fazio & Olson, 2003). A further limitation concerns the study’s sample size Although the sample of 85 participants was larger than the minimum required sample size of *N* = 82 according to the statistical power analysis, it can still be considered small for a psychometric study. Finally, this study did not examine the test-retest reliability of the ST-IAT, which is important for demonstrating the instrument’s stability over time as part of its psychometric validity.

Future research is recommended to replicate the findings of the present study and previous research into the reliability and validity of both single- and dual-target IATs, including head-to-head comparisons of the different IAT versions, as well as an investigation of the ST-IAT’s test-retest reliability.

## Conclusion

The present study provided preliminary support for the reliability and validity of the Transgender ST-IAT. Pending replication of the present findings in new samples, the findings imply that the Transgender ST-IAT can be used with confidence in research into implicit attitudes toward transgender individuals.

## Declaration of competing interest

The authors declare that there are no competing interests.

## References

[bib0001] Axt J.R., Conway M.A., Westgate E.C., Buttrick N.R. (2021). Implicit transgender attitudes independently predict beliefs about gender and transgender people. Personality and Social Psychology Bulletin.

[bib0002] Ballinger T., Canevello A., Crocker J., Jiang T., Quinn D.M. (2023). Self-disclosure of concealable stigmatized identities: A dyadic longitudinal investigation guided by the contact hypothesis. Journal of Social Issues.

[bib0003] Barbir L.A., Vandevender A.W., Cohn T.J. (2017). Friendship, attitudes, and behavioral intentions of cisgender heterosexuals toward transgender individuals. Journal of Gay & Lesbian Mental Health.

[bib0004] Bargh J.A., Wyer R.S., Srull T.K. (1994). Handbook of social cognition, vol. 1: Basic processes; vol. 2: Applications (2nd ed.).

[bib0005] Beaton D.E., Bombardier C., Guillemin F., Ferraz M.B. (2000). Guidelines for the process of cross-cultural adaptation of self-report measures. Spine (Phila Pa 1976).

[bib0006] Belzak W.C.M., Lockwood J.R. (2024). Estimating test-retest reliability in the presence of self-selection bias and learning/practice effects. Applied Psychological Measurement.

[bib0007] Bluemke M., Friese M. (2008). Reliability and validity of the single-Target IAT (ST-IAT): Assessing automatic affect towards multiple attitude objects. European Journal of Social Psychology.

[bib0008] Bos A.E.R., Pryor J.B., Reeder G.D., Stutterheim S.E. (2013). Stigma: Advances in theory and research. Basic and Applied Social Psychology.

[bib0009] Burguet A., Girard F. (2024). Stigma of schizophrenia and bipolar disorders: Explicit and implicit measures among mental health professionals. Stigma and Health.

[bib0010] Burke S.E., Jaurique A., Valen B.M., Wittlin N.M., McDonald M.L., LaFrance M. (2026). Forms of psychological bias against transgender women and men and people with nonbinary gender identities. Personality and Social Psychology Review.

[bib0011] Carmines E., Nassar R. (2021). How social desirability bias affects immigration attitudes in a hyperpolarized political environment. Social Science Quarterly.

[bib0012] Chinazzo Í.R., Vaitses Fontanari A.M., Riva A., de Brito Silva B., Rodrigues L.P., Trajano A.C., Feijó M., Brandelli Costa A., Lobato M.I.R. (2025). Coping strategies employed by transgender youth with higher and lower quality of life. British Journal of Developmental Psychology.

[bib0013] Coleman E., Radix A.E., Bouman W.P., Brown G.R., de Vries A.L.C., Deutsch M.B., Ettner R., Fraser L., Goodman M., Green J., Hancock A.B., Johnson T.W., Karasic D.H., Knudson G.A., Leibowitz S.F., Meyer-Bahlburg H.F.L., Monstrey S.J., Motmans J., Nahata L., Arcelus J. (2022). Standards of care for the health of transgender and gender diverse people, version 8. International Journal Of Transgender Health.

[bib0014] Coleman J.M., Hong Y.-Y. (2008). Beyond nature and nurture: The influence of lay gender theories on self-stereotyping. Self and Identity.

[bib0015] Corr P.J. (2010). Automatic and controlled processes in behavioural control: Implications for personality psychology. European Journal of Personality.

[bib0016] Dasgupta N., McGhee D.E., Greenwald A.G., Banaji M.R. (2000). Automatic preference for White Americans: Eliminating the familiarity explanation. Journal of Experimental Social Psychology.

[bib0017] Davies K., Tropp L.R., Aron A., Pettigrew T.F., Wright S.C. (2011). Cross-group friendships and intergroup attitudes: A meta-analytic review. Personality and Social Psychology Review.

[bib0018] Dawson S.J., Huberman J.S., Bouchard K.N., McInnis M.K., Pukall C.F., Chivers M.L. (2019). Effects of individual difference variables, gender, and exclusivity of sexual attraction on volunteer bias in sexuality research. Archives Of Sexual Behavior.

[bib0019] De Houwer J., Teige-Mocigemba S., Spruyt A., Moors A. (2009). Implicit measures: A normative analysis and review. Psychological Bulletin.

[bib0020] Derbyshire D.W., Keay T. (2023). Nurses' implicit and explicit attitudes towards transgender people and the need for trans-affirming care. Heliyon.

[bib0021] Dewitte M., De Houwer J., Buysse A. (2008). The role of automatic approach-avoidance tendencies in adult attachment. International Journal of Psychology.

[bib0022] Dierckx, M., Motmans, J., Meier, P., Dieleman, M., & Pezeril, C. (2014). Attitudemeting met betrekking tot seksisme, holebifobie en transfobie: “beyond the box".

[bib0023] Drabish K., Theeke L.A. (2022). Health impact of stigma, discrimination, prejudice, and bias experienced by transgender people: A systematic review of quantitative studies. Issues in Mental Health Nursing.

[bib0024] Dunlap W.P., Burke M.J., Greer T. (1995). The effect of skew on the magnitude of product-moment correlations. Journal of General Psychology.

[bib0025] Ellwart T., Konradt U. (2011). Formative versus reflective measurement: An illustration using work-family balance. Journal of Psychology.

[bib0026] Erkal E., Kiyak E., Uren Y., Milanlioglu A. (2024). Determination of stigma and attitude in relatives of patients with epilepsy. Seizure: European Journal of Epilepsy.

[bib0027] Esacove A. (2024). Common patterns of cisgender use in public health articles and their implications for gender inclusivity efforts, 2013‒2020. American Journal of Public Health.

[bib0028] Fazio R.H., Olson M.A. (2003). Implicit measures in social cognition research: Their meaning and use. Annual Review of Psychology.

[bib0029] Frost D.M., Meyer I.H. (2023). Minority stress theory: Application, critique, and continued relevance. Current Opinion in Psychology.

[bib0030] Gawronski B., Payne B.K. (2010). https://research.ebsco.com/linkprocessor/plink?id=0951789c-2334-3990-beb4-d079f56f84ab.

[bib0031] Greenwald A.G., Brendl M., Cai H., Cvencek D., Dovidio J.F., Friese M., Hahn A., Hehman E., Hofmann W., Hughes S., Hussey I., Jordan C., Kirby T.A., Lai C.K., Lang J.W.B., Lindgren K.P., Maison D., Ostafin B.D., Rae J.R., Wiers R.W. (2022). Best research practices for using the implicit association test. Behavior Research Methods.

[bib0032] Greenwald A.G., McGhee D.E., Schwartz J.L. (1998). Measuring individual differences in implicit cognition: The implicit association test. Journal of Personality and Social Psychology.

[bib0033] Greenwald A.G., Poehlman T.A., Uhlmann E.L., Banaji M.R. (2009). Understanding and using the implicit association test: III meta-analysis of predictive validity. Journal of Personality and Social Psychology.

[bib0034] Gruijters, S., Fleuren, B., & Peters, G.-J. (2021). Crossing the seven Cs of internal consistency: Assessing the reliability of formative instruments. PsyArXiv. 10.31234/osf.io/qar39.

[bib0035] Gülgöz S., DeMeules M., Gelman S.A., Olson K.R. (2019). Gender essentialism in transgender and cisgender children. PLoS One.

[bib0036] Hatch H.A., Warner R.H., Broussard K.A., Harton H.C. (2022). Predictors of transgender prejudice: A meta-analysis. Sex Roles.

[bib0037] Hatzenbuehler M.L., Pachankis J.E. (2016). Stigma and minority stress as social determinants of health among Lesbian, Gay, Bisexual, and transgender youth: Research evidence and clinical implications. Pediatric Clinics of North America.

[bib0038] Hofmann W., Gawronski B., Gschwendner T., Le H., Schmitt M. (2005). A meta-analysis on the correlation between the implicit association test and explicit self-report measures. Personality and Social Psychology Bulletin.

[bib0039] Houben K., Wiers R.W. (2008). Measuring implicit alcohol associations via the internet: Validation of web-based implicit association tests. Behavior Research Methods, Instruments, & Computers.

[bib0040] Maniaci G., La Cascia C., Sartorio R.C., Ferraro L., Giammanco A., Anselmo R., Oddo G., Parrinello E., Toia F., Zabbia G., Cordova A., La Barbera D. (2022). Alterations in body image, self-esteem and quality of life in a sample of Italian transgender individuals before gender- affirming surgery. Journal of Psychopathology.

[bib0041] McNally R.J. (1995). Automaticity and the anxiety disorders. Behaviour Research and Therapy.

[bib0042] McNulty J.K., Baker L.R., Olson M.A. (2014). Implicit self-evaluations predict changes in implicit partner evaluations. Psychological Science.

[bib0043] Mechler C., Scheer D., Heyl V. (2024). Reliability of a shortened single-target implicit association test (ST-IAT) as an implicit measure of attitudes towards inclusion. Replication and extension of “implicitly measuring attitudes towards inclusive education: A new attitude test based on single-target implicit associations” (Lüke and Grosche, 2018, European journal of special needs education). European Journal of Special Needs Education.

[bib0044] Meyer I.H. (2003). Prejudice, social stress, and mental health in lesbian, gay, and bisexual populations: Conceptual issues and research evidence. Psychological Bulletin.

[bib0045] Morris A., Kurdi B. (2023). Awareness of implicit attitudes: Large-scale investigations of mechanism and scope. Journal of Experimental Psychology: General.

[bib0046] Nosek B.A. (2005). Moderators of the relationship between implicit and explicit evaluation. Journal of Experimental Psychology: General.

[bib0047] Nosek B.A. (2007). Implicit-explicit relations. Current Directions in Psychological Science.

[bib0048] Perez-Arche H., Miller D.J. (2021). What predicts attitudes toward transgender and nonbinary people? An exploration of gender, authoritarianism, social dominance, and gender ideology. Sex Roles.

[bib0049] Pettigrew T.F. (1997). Generalized intergroup contact effects on prejudice. Personality and Social Psychology Bulletin.

[bib0050] Schober P., Boer C., Schwarte L.A. (2018). Correlation coefficients: Appropriate use and interpretation. Anesthesia & Analgesia.

[bib0051] Shiffrin R.M., Schneider W. (1977). Controlled and automatic human information processing: II. Perceptual learning, automatic attending and a general theory. Psychological Review.

[bib0052] Tee N., Hegarty P. (2006). Predicting opposition to the civil rights of Trans persons in the United Kingdom. Journal of Community & Applied Social Psychology.

[bib0053] Tsai T.I., Luck L., Jefferies D., Wilkes L. (2018). Challenges in adapting a survey: Ensuring cross-cultural equivalence. Nurse Researcher.

[bib0054] Walch S.E., Ngamake S.T., Francisco J., Stitt R.L., Shingler K.A. (2012). The attitudes toward transgendered individuals scale: Psychometric properties. Archives of Sexual Behavior.

[bib0055] Walch S.E., Sinkkanen K.A., Swain E.M., Francisco J., Breaux C.A., Sjoberg M.D. (2012). Using intergroup contact theory to reduce stigma against transgender individuals: Impact of a transgender speaker panel presentation. Journal of Applied Social Psychology.

[bib0056] Wang-Jones T.T.S., Alhassoon O.M., Hattrup K., Ferdman B.M., Lowman R.L. (2017). Development of gender identity implicit association tests to assess attitudes toward transmen and transwomen. Psychology of Sexual Orientation and Gender Diversity.

[bib0057] Wang-Jones T.T.S., Hauson A.O., Ferdman B.M., Hattrup K., Lowman R.L. (2018). Comparing implicit and explicit attitudes of gay, straight, and non-monosexual groups toward transmen and transwomen. International Journal of Transgenderism.

[bib0058] White Hughto J.M., Reisner S.L., Pachankis J.E. (2015). Transgender stigma and health: A critical review of stigma determinants, mechanisms, and interventions. Social Science & Medicine.

[bib0059] Wigboldus, D.H., Holland, R.W., & van Knippenberg, A. (2004). Single target implicit associations. Unpublished manuscript, 265(11).

[bib0060] Wilton L.S., Bell A.N., Carpinella C.M., Young D.M., Meyers C., Clapham R. (2019). Lay theories of gender influence support for women and transgender people’s legal rights. Social Psychological and Personality Science.

[bib0061] Yamaguchi S., Ando S., Nishida A., Kasai K., Koike S. (2025). Contact experiences of adolescents and family members are associated with decrease of personal stigma but increase of perceived stigma. Journal of Adolescence.

[bib0062] Zapata-Calvente A.L., Moya M., Bohner G., Megías J.L. (2019). Automatic associations and conscious attitudes predict different aspects of men’s intimate partner violence and sexual harassment proclivities. Sex Roles.

